# Psychometric Validation of the Parental Bonding Instrument in a U.K. Population–Based Sample: Role of Gender and Association With Mental Health in Mid-Late Life

**DOI:** 10.1177/1073191116660813

**Published:** 2016-08-01

**Authors:** Man K. Xu, Alexandre J. S. Morin, Herbert W. Marsh, Marcus Richards, Peter B. Jones

**Affiliations:** 1 Leiden University Medical Center, Leiden, Netherlands; 2 Department of Psychiatry, University of Cambridge, Cambridge, UK; 3 Australian Catholic University, Sydney, New South Wales, Australia; 4 King Saud University; 5 University College London, London, UK

**Keywords:** parenting, adolescence, Parental Bonding Instrument, psychometrics, measurement invariance, birth cohort, structural equation modeling

## Abstract

The factorial structure of the Parental Bonding Instrument (PBI) has been frequently studied in diverse samples but no study has examined its psychometric properties from large, population-based samples. In particular, important questions have not been addressed such as the measurement invariance properties across parental and offspring gender. We evaluated the PBI based on responses from a large, representative population-based sample, using an exploratory structural equation modeling method appropriate for categorical data. Analysis revealed a three-factor structure representing “care,” “overprotection,” and “autonomy” parenting styles. In terms of psychometric measurement validity, our results supported the complete invariance of the PBI ratings across sons and daughters for their mothers and fathers. The PBI ratings were also robust in relation to personality and mental health status. In terms of predictive value, paternal care showed a protective effect on mental health at age 43 in sons. The PBI is a sound instrument for capturing perceived parenting styles, and is predictive of mental health in middle adulthood.

Developmental theories of attachment (Bowlby, 1977) and emotion regulation (Sroufe, 1997) maintain that parenting practices influence various aspects of children’s emotional and behavioral development. Positive parenting practices (e.g., care, responsiveness, and clear standards; Baumrind, 1991a) are associated with many desirable outcomes such as better adjustment (Thorberg, Young, Sullivan, & Lyvers, 2011), prosocial dispositions (Collins & Steinberg, 2006), and lower risk for the development of internalizing (Heider, Matschinger, Bernert, Alonso, & Angermeyer, 2006) and externalizing (Baumrind, 1991b) symptoms, as well as eating (Swanson et al., 2010) and addictive (Siomos et al., 2012) disorders. Negative parenting practices (e.g., a high level of parental control and a low level of autonomy and warmth; Baumrind, 1967, 1971) are associated with ill adjustment (Rodgers, 1996a, 1996b; Thorberg et al., 2011) including low self-esteem (Milevsky, Schlechter, Netter, & Keehn, 2007), gambling addiction (Villalta, Arévalo, Valdepérez, Pascual, & de los Cobos, 2015), and social hostility (Ladd & Pettit, 2002), as well as worse later life well-being (Huppert, Abbott, Ploubidis, Richards, & Kuh, 2009). The assessment of parenting for very young children is typically conducted through observation. However, for the assessment of parenting style by older children and adults, offspring self-reports are more often used. The Parental Bonding Instrument (PBI) is a popular measurement tool for this purpose. The PBI retrospectively measures participants’ perceptions of their relationship with their parents before the age of 16 years, and can be administered retrospectively at any age after the age 16. The instrument assesses these self-perceived relationships separately for the mother and the father, through two dimensions of parenting: care and control (Parker, Tupling, & Brown, 1979). The care dimension measures positive parenting, including parental warmth and affection. The control dimension measures negative parenting, including parental control and constraint.

## Psychometric Properties of the PBI and Methodological Challenges

Despite its widespread use, there is no consensus regarding the factor structure of the PBI. While some studies have confirmed the original two-factor structure (Kitamura et al., 2009; Mackinnon, Henderson, Scott, & Duncan-Jones, 1989; Parker et al., 1979), other studies have suggested three- (Cox, Enns, & Clara, 2000; Cubis, Lewin, & Dawes, 1989; Heider et al., 2005; E. Murphy, Brewin, & Silka, 1997; Sato et al., 1999) or four-factor solutions (Behzadi & Parker, 2015; Liu, Li, & Fang, 2011; Uji, Tanaka, Shono, & Kitamura, 2006). In most previous studies converging on a three-factor solution, items within the control factor have been shown to form two distinct factors: (a) overprotection, consisting of items such as “[my mother/father] felt I could not look after myself unless she or he was around” and (b) autonomy, consisting of items such as “[my mother/father] let me decide things for myself.” In four-factor solutions, items originally measuring the care factor also separated into two dimensions, although this was observed mainly in non-European samples of Japanese (Uji et al., 2006), Chinese (Liu et al., 2011), and Persian respondents (Behzadi & Parker, 2015).

Apart from cultural or linguistic differences, several methodological issues may explain these factor structure inconsistencies. While some studies relied on exploratory factor analytic (EFA) methods or principal component analyses (e.g., Gómez-Beneyto, Pedrós, Tomás, Aguilar, & Leal, 1993; E. Murphy et al., 1997), others utilized confirmatory factor analyses (CFAs, e.g., Behzadi & Parker, 2015; Terra et al., 2009; Tsaousis, Mascha, & Giovazolias, 2011). Although traditional EFA methods can be useful for determining the number of factors to retain, they typically do not provide goodness-of-fit information; it is therefore difficult to assess whether the model provides an adequate representation of the data. On the other hand, although CFA methods are able to test theory-driven models and provide goodness-of-fit information, these methods rely on strict assumptions that do not often hold in practice. For instance, CFA relies on the highly restrictive independent cluster assumption, which forces all cross-loadings to be zero. When nonzero cross-loadings are present in the population, such constraints can inflate the degree of associations between factors (Marsh, Morin, Parker, & Kaur, 2013; Morin, Marsh, & Nagengast, 2013). An increasingly popular method, exploratory structural equation modeling (ESEM), combines features of CFA and EFA, thus overcoming the typical restrictions of both and allowing the free estimation of all possible cross-loadings between items and nontarget factors (Marsh et al., 2013; Morin et al., 2013). The main advantage of ESEM over CFA is that it integrates the less restrictive assumptions of EFA with the benefits of structural equation modeling, such as goodness-of-fit indices, multigroup invariance analyses, and the ability to combine regression and structural equations within the same model (Asparouhov & Muthén, 2009; Marsh et al., 2009; Marsh et al., 2010; Marsh, Nagengast, & Morin, 2012). Furthermore, simulations studies and studies of simulated data showed that ESEM tends to provide more exact estimates of true population values for factor correlations when cross-loadings are present in the population model, and to remain unbiased when the population model corresponds to the CFA assumption (Asparouhov, Muthén, & Morin, 2015).

A large body of empirical research (X. Chen, Liu, & Li, 2000; Cubis et al., 1989; Henry, Tolan, & Gorman-Smith, 2005; Hoeve, Dubas, Gerris, van der Laan, & Smeenk, 2011; Lansford, Laird, Pettit, Bates, & Dodge, 2014; Watson, Potts, Hardcastle, Forehand, & Compas, 2012) suggests that the association between parenting practices and offspring outcomes is dependent on the gender of the parent and of the offspring. Consistency of parenting style between both parents has also been investigated (Winsler, Madigan, & Aquilino, 2005). However, an important prerequisite to these comparisons is the demonstration that the PBI factors are psychometrically invariant across males and female offspring’s ratings of their mothers and fathers (Byrne, Shavelson, & Muthén, 1989; Cheung, 2008). To our knowledge, no studies have examined the measurement invariance properties of the PBI instrument in relation to the gender of the offspring as well as of the parents.

As with many other instruments relying on self-reported measures, PBI ratings have been shown to be influenced by current depressive states (Eleanor Murphy, Wickramaratne, & Weissman, 2010) or sad mood (Gillham, Putter, & Kash, 2007). Similarly, personality has also been suggested to represent a possible source of bias in PBI ratings due to the subjective ways in which items are interpreted, which itself can be influenced by various respondent personality characteristics (Jakobsen & Jensen, 2015; Randall & Fernandes, 1991). Psychometric methods, such as multiple indicators multiple causes (MIMIC) tests of differential item functioning (DIF), can address this issue by detecting the extent to which item response differs as a function of various characteristics of the respondents over and above the relations between these characteristics and scores on the PBI factors. To our knowledge, no such studies have formally studied the response bias of the PBI in relation to personality or depressive state using state-of-the-art psychometric methods.

Furthermore, the PBI items are rated using a 4-point, ordered–categorical, Likert-type response scale with a marked tendency toward nonnormality (Liu et al., 2011; Tsaousis et al., 2011). Under these conditions, research has shown that it is problematic to model data using an estimator (such as maximum likelihood or robust alternative) that assumes the underlying continuity of the ratings (DiStefano, 2002; Dolan, 1994). To date, no psychometric study of the PBI has properly taken into account the ordinal nature of the PBI items using an estimation method that model data as ordinal variables such as the robust weighted least squares estimator (WLSMV; Asparouhov & Muthén, 2010; Finney & DiStefano, 2006).

## Predictive Validity of the PBI

Although the PBI has been recognized to be predictive of future behavioral outcomes, many previous studies exploring parenting effects focus on young children (Cooper-Vince, Chan, Pincus, & Comer, 2014; Möller, Majdandžić, & Bögels, 2015; StGeorge, Fletcher, Freeman, Paquette, & Dumont, 2015) or adolescents (Lansford et al., 2014; McKinney & Renk, 2008). Few studies have followed participants into adulthood (Hoeve et al., 2011). Another weakness of previous investigations of the PBI is that very few have been conducted based on population-representative samples, thus rendering findings vulnerable to selection bias. In the present investigation, we relied on a longitudinal population-based sample from England, Wales, and Scotland to assess the psychometric properties of the PBI and its measurement invariance in relation to the gender of offspring and the parents, using multiple-group ESEM. Specifically, we assessed two psychometric properties of the PBI, as well as its predictive validity. Regarding the psychometric properties of the PBI, we address the following questions:

1. How many factors are necessary to represent PBI ratings, as assessed by ESEM analyses conducted separately for maternal and paternal PBI ratings?
2. Is the PBI underlying measurement model invariant for ratings provided by male and female offspring of the parenting style of their mothers and fathers?
3. Are PBI ratings biased (DIF) as a function of respondents’ personality (measured at age 26) and mental health status (measured at age 43)?

Regarding the predictive validity of the PBI, we address the following question:

What is the unique predictive effect of maternal or paternal PBI factors on respondents’ mental health assessed at age 43 and 53?

## Method

### Sample

The study sample was based on the Medical Research Council (MRC) National Survey of Health and Development (NSHD), also known as the British 1946 birth cohort, which originally consisted of 5,362 singleton babies (2,547 girls and 2,815 boys) born in 1 week in March 1946 in England, Scotland, and Wales (Stafford et al., 2013). Study members were all classified as White. The data collection received approval from the North Thames Multi-Centre Research Ethics Committee, and participants gave informed consent to participate in the MRC NSHD.

### Measures

#### Occupational Social Class

The socioeconomic status of respondents during their childhood was assessed via the father’s occupational social class (based on the U.K. Registrar General’s classification) when study members were aged 11 years. If this information was missing, it was replaced by similar information obtained at age 4 or 15, depending on availability. The adulthood occupational social class was measured at 43 years. If this information was missing, it was replaced by similar information obtained at age 36 or 26. The occupational social class variable has six categories (1: Unskilled, 2: Partly Skilled, 3: Skilled [Manual], 4: Skilled [Nonmanual], 5: Intermediate, 6: Professional).

#### The Parental Bonding Instrument

At age 43, the survey members rated their mothers’ (24 items) and fathers’ (24 items) parenting practices for the period up to the age of 16 years. These items were rated on a 1 to 4 ordered–categorical, Likert-type scale ranging from *very like this* to *very unlike this*. See Table 2 for a list of the PBI items.

#### Maudsley Personality Inventory

Study members completed six Neuroticism (e.g., “Do you sometimes feel happy, sometimes depressed, without any apparent reason?”) and six Extraversion (e.g., “Are you happiest when you get involved in some project that calls for rapid action?”) items from the Maudsley Personality Inventory (Eysenck, 1958, 1959) at age 26. The items had a binary response format of “no” and “yes.” Kuder–Richardson 20 (the equivalent of Cronbach’s α for binary items) scale score reliability coefficients are 0.554 for extraversion and 0.741 for neuroticism.

#### Psychiatric Symptom Frequency Scale

Anxiety and depression at age 43 were assessed through the interview-based Psychiatric Symptom Frequency scale (Lindelow, Hardy, & Rodgers, 1997). Participants provided ratings ranging from 0 (*not in the past year*) to 5 (*very often*) to 18 questions such as “Have you felt on edge or keyed up or mentally tense?” in the past 12 months (α = 0.896).

#### General Health Questionnaire

Participants completed the 28-item self-administered General Health Questionnaire (Goldberg & Hillier, 1979) at age 53. The 28-item General Health Questionnaire focuses on symptoms of anxiety and depression in the preceding 4 weeks (e.g., “Have you recently been getting scared or panicky for no good reason?”). Item data were coded on a 4-point Likert-type scale ranging from *not at all* to *much more than usual* (α = 0.926).

### Statistical Analysis

Analyses were carried out using M*plus* 7.11 (Muthén & Muthén, 2015). We used the WLSMV estimator with theta parameterization. Data from questionnaire items were modelled as ordered–categorical polytomous ratings through a probit regression link with the corresponding latent variables. This corresponds to a graded response, two-parameter, normal ogive model in item response theory terms (Samejima, 1997).

We used ESEM to determine whether the PBI data structure was invariant across groups formed on the basis of the gender of the offspring (sons and daughters) and their parents (mothers and fathers). Analyses started with the estimation of ESEMs using oblique geomin rotation, with an epsilon value of 0.5 (Marsh et al., 2009; Morin et al., 2013). Items related to maternal and paternal parenting styles were analyzed separately in order to determine the number of factors to retain, and to examine whether the factor structure was comparable across maternal and paternal measures. Since the wording of the items is identical for maternal and paternal ratings, a priori correlated residuals at the item level were included between items with parallel wording, as recommended by Marsh and Hau (1996). Next, measurement invariance was examined using multiple-group ESEM (Marsh et al., 2013; Meredith & Teresi, 2006) for configural invariance, weak invariance (factor loadings), strong invariance (loadings and thresholds), and strict invariance (loadings, thresholds, and uniquenesses). Although it was not strictly part of the measurement invariance assessment, we also assessed the structural invariance of the PBI in terms of factor variances, covariances, and latent means across groups.

To assess DIF in relation to affective symptoms and personality measures, we used a MIMIC ESEM (Morin et al., 2013). DIF represents a direct association between the covariate and a particular item after accounting for the association between the covariate and the latent factor, which indicates that the covariate influences the response process on a particular item over and above its influence on the latent factor itself. DIF is thus similar to a case of threshold noninvariance across levels of the covariate, and suggests the presence of response bias at the item level (Kaplan, 2000; Morin et al., 2013). Specifically, three models are tested in a MIMIC analysis. In the MIMIC saturated model, the paths from the covariates to the latent factors are fixed at zero, but all direct paths from the covariates to the items are estimated. In the MIMIC invariant model, the paths from the covariates to the items are fixed at zero, but the paths from the covariates to the latent factors are freely estimated. In the third MIMIC Null model, all paths from the covariates to the latent factors and items are constrained to be zero. A goodness-of-fit comparison between the first two models (Invariant and Saturated) and the last (Null) serves to assess whether the covariates have an effect on PBI ratings, whereas the comparison between the first two models (Invariant vs. Saturated) serves to assess the presence of DIF.

Since the chi-square is known to be highly sensitive to sample size (Marsh, Balla, & McDonald, 1988; Marsh, Hau, & Grayson, 2005), a variety of sample size independent goodness-of-fit indices was also examined to assess the fit of the alternative models: the root mean square error of approximation (RMSEA), the Tucker–Lewis index (TLI), and the comparative fit index (CFI; Fan, Thompson, & Wang, 1999; Hu & Bentler, 1999; Marsh, Hau, & Wen, 2004; Yu, 2002). The TLI and CFI vary along a 0 to 1 continuum and values greater than 0.90 and 0.95 typically reflect an acceptable and excellent fit to the data. RMSEA values of less than 0.06 and 0.08 indicate a close fit and an acceptable fit to the data, respectively. In terms of model comparisons for multiple-group analyses, a restrictive model is preferred if the change in model fit indices is not significantly inferior to those of the less restrictive model. For RMSEA, the change should be less than 0.015 (F. Chen, 2007). For CFI and TLI, the change should be less than 0.01 (F. Chen, 2007; Cheung & Rensvold, 2001). The WLSMV chi-square difference tests (computed with the DIFFTEST function, Muthén & Muthén, 2015) compare the model under investigation to less restrictive alternative model.

## Results

### Sample Demographics

Of the initial 5,362 newborn babies, 2,815 were male and 2,547 were female. For the main variables used in the current analysis (parental bonding at age 43, personality at age 26, and mental health data at ages 43 and 35), there were 1,217 participants with complete data, whereas information was completely missing for 1,373 participants. In comparison to the participants with complete data, the samples with completely missing data had a higher percentage of males, and came from families of lower occupational social class at age 11 and had lower occupational social class at age 43.

### Psychometric Properties

The results of PBI psychometric properties are presented in relation to number of PBI factors, measurement invariance according to gender, and uniform DIF in relation to personality and mental health measures.

#### Number of PBI Factors

Mother- and father-specific PBI items were separately analyzed using ESEM. Models including two, three, and four factors were compared, and the results showed that ESEMs including three or four factors provided a satisfactory level of fit to the data (Table 1). Parameter estimates from these models are reported in Tables 2 (mothers) and 3 (fathers). These results show that, in both models, Factor 1 describes the a priori “care” dimension of the PBI questionnaire, whereas Factors 2 and 3 describe the “overprotection” and “autonomy” dimensions, whose items jointly form the original “control” factor. In both maternal and paternal measures, the “care” factor was negatively correlated with “overprotection” factor but positively correlated with the “autonomy” factor, whereas the “overprotection” factor was negatively correlated with the “autonomy” factor. However, the four-factor solution was not fully equivalent across ratings of mothers and fathers. For ratings of fathers, the fourth factor merely corresponded to a single item (“wanted me to grow up”) from the “overprotection” factor. In contrast, in ratings of the mothers, the “overprotection” factor was more cleanly split into two factors. A close examination revealed that the three items corresponding to the third factor in the rating of the mothers had largely parallel wording (i.e., “gave me as much freedom as I wanted,” “let me go out as often as I wanted,” and “let me dress in any way I pleased”). We thus included correlated residuals between these three items and reran the analyses for the three-factor model. This revised three-factor model, including correlated residuals, provided a very clear three-factor solution, with similar solutions across ratings of mothers and fathers, and corresponded to the a priori “care,” “overprotection,” and “autonomy” factors found in many previous studies. This factor structure fits the data well and is consistent across ratings of the mothers and fathers. Subsequent analyses are therefore based on this factor structure.

**Table 1.** Summary of Goodness-of-Fit Statistics for Whole Sample ESEM.

	Mother measures, *n* = 3,175					Father measures, *n* = 3,071				
	χ^2^	*df*	RMSEA	CFI	TLI	χ^2^	*df*	RMSEA	CFI	TLI
Two-factor ESEM	11368.038	229	0.124	0.862	0.833	12069.940	229	0.130	0.895	0.873
Three-factor ESEM	3939.085	207	0.075	0.954	0.938	4269.616	207	0.080	0.964	0.952
Four-factor ESEM	2337.030	186	0.060	0.973	0.960	2606.764	186	0.065	0.979	0.968
Three-factor ESEM, CU	3082.005	204	0.067	0.964	0.952	3641.108	204	0.074	0.970	0.959
Four-factor ESEM, CU	2086.701	183	0.057	0.976	0.964	2170.735	183	0.059	0.982	0.973

*Note*. ESEM = exploratory structural equation modeling; *df* = degrees of freedom; RMSEA = root mean square error of approximation; CFI = comparative fit index; TLI = Tucker–Lewis index; CU = correlated residuals. Residual variances were specified for three items: “gave me as much freedom as I wanted,” “let me go out as often as I wanted,” and “let me dress in any way I pleased.”

**Table 2.** Factor Loadings From the Exploratory Structural Equation Models for Maternal Measures.

	Mother measure factor structure									
	Three factor			Four factor				Three factor with correlated residuals^a^		
	Factor 1	Factor 2	Factor 3	Factor 1	Factor 2	Factor 3	Factor 4	Care	Overprotection	Autonomy
Spoke to me with a warm and friendly voice	**0.842**	−0.007	−0.021	**0.830**	−0.068	0.014	−0.033	**0.817**	−0.034	0.019
My father helped me as much as I needed	**0.872**	0.031	−0.015	**0.856**	−0.013	−0.001	0.001	**0.832**	0.013	0.046
Appeared to understand my problems and worries	**0.832**	−0.012	0.084	**0.816**	0.025	0.030	0.168	**0.735**	−0.002	0.208
Was affectionate to me	**0.867**	0.081	0.012	**0.849**	0.022	0.033	−0.029	**0.830**	0.052	0.056
Enjoyed talking things over with me	**0.792**	0.052	0.004	**0.777**	0.075	−0.047	0.128	**0.729**	0.052	0.097
Frequently smiled at me	**0.764**	0.018	−0.025	**0.751**	−0.042	0.007	−0.043	**0.763**	−0.022	−0.025
Seemed to understand what I needed or wanted	**0.769**	−0.028	0.065	**0.755**	0.018	0.007	0.178	**0.681**	−0.017	0.183
Made me feel I was not wanted	**−0.471**	0.325	0.156	**−0.486**	**0.375**	0.075	0.047	**−0.556**	**0.373**	0.230
Could make me feel better when I was upset	**0.785**	0.006	−0.011	**0.773**	−0.026	−0.004	0.018	**0.763**	−0.020	0.021
Talked to me often	**0.803**	−0.008	−0.034	**0.792**	−0.042	−0.024	0.017	**0.798**	−0.044	−0.022
Praised me	**0.766**	0.080	0.027	**0.751**	0.008	0.059	−0.067	**0.740**	0.048	0.048
Let me do those things I liked doing	**0.511**	−0.015	**0.350**	**0.490**	−0.005	**0.337**	0.091	0.342	0.006	**0.502**
Tried to control everything I did	−0.057	**0.638**	−0.088	−0.082	**0.681**	−0.228	0.052	−0.108	**0.666**	−0.013
Invaded my privacy	−0.127	**0.663**	−0.035	−0.157	**0.680**	−0.156	0.009	−0.184	**0.692**	0.034
Tended to baby me	0.269	**0.753**	0.038	0.234	**0.551**	0.089	−0.408	0.271	**0.715**	−0.011
Tried to make me dependent on him	0.044	**0.711**	0.013	0.013	**0.602**	−0.007	−0.238	0.009	**0.711**	0.033
Felt I could not look after myself unless he was around	−0.006	**0.760**	0.004	−0.037	**0.601**	0.022	−0.350	−0.013	**0.748**	−0.016
Was overprotective of me	0.232	**0.747**	−0.098	0.204	**0.545**	−0.051	−0.415	0.268	**0.715**	−0.153
Liked me to make my own decisions	**0.310**	−0.253	**0.403**	0.283	0.008	0.241	**0.571**	0.003	−0.130	**0.761**
Wanted me to grow up	0.121	−0.082	**0.213**	0.099	0.181	0.019	**0.531**	−0.097	0.021	**0.484**
Let me decide things for myself	0.271	−0.302	**0.389**	0.254	−0.126	0.287	**0.408**	0.036	−0.227	**0.636**
Gave me as much freedom as I wanted^a^	0.002	−0.020	**0.868**	−0.021	−0.029	**0.866**	0.038	−0.010	−0.111	**0.571**
Let me go out as often as I wanted^a^	−0.017	0.066	**0.904**	−0.046	0.031	**0.915**	−0.022	0.006	−0.047	**0.532**
Let me dress in ZZany way I pleased^a^	0.121	0.029	**0.629**	0.098	0.013	**0.635**	0.014	0.065	−0.019	**0.485**
	Factor correlation									
	Factor 1	Factor 2	Factor 3	Factor 1	Factor 2	Factor 3	Factor 4	Care	Overprotection	Autonomy
Factor 1 (care)	1.000			1.000				1.000		
Factor 2 (overprotection)	−0.291	1.000		−0.133	1.000			−0.228	1.000	
Factor 3 (autonomy)	0.408	−0.317	1.000	0.415	−0.165	1.000		0.591	−0.372	1.000
Factor 4				0.222	−0.230	0.337	1.000			

*Note*. Values in bold are to suggest the corresponding factors.

a Residual variances were specified for three items: “gave me as much freedom as I wanted,” “let me go out as often as I wanted,” and “let me dress in any way I pleased.”

**Table 3.** Factor Loadings From the Exploratory Structural Equation Models for Paternal Measures.

	Father measure factor structure									
	Three factor			Four factor				Three factor with correlated residuals^a^		
	Factor 1	Factor 2	Factor 3	Factor 1	Factor 2	Factor 3	Factor 4	Care	Overprotection	Autonomy
Spoke to me with a warm and friendly voice	**0.838**	−0.011	0.054	**0.833**	−0.036	0.077	−0.202	**0.802**	−0.062	0.083
My father helped me as much as I needed	**0.836**	−0.011	0.047	**0.846**	−0.008	0.019	0.038	**0.779**	−0.017	0.129
Appeared to understand my problems and worries	**0.820**	0.008	0.100	**0.834**	0.019	0.034	0.204	**0.735**	0.028	0.218
Was affectionate to me	**0.867**	0.040	0.003	**0.864**	0.023	0.017	−0.155	**0.845**	−0.013	0.025
Enjoyed talking things over with me	**0.872**	0.029	−0.041	**0.888**	0.029	−0.097	0.122	**0.851**	−0.006	0.001
Frequently smiled at me	**0.815**	−0.002	−0.005	**0.810**	−0.024	0.016	−0.196	**0.803**	−0.063	0.000
Seemed to understand what I needed or wanted	**0.795**	0.008	0.113	**0.808**	0.022	0.043	0.233	**0.708**	0.029	0.229
Made me feel I was not wanted	**−0.407**	**0.422**	0.083	**−0.414**	**0.441**	0.072	0.160	**−0.434**	**0.469**	0.109
Could make me feel better when I was upset	**0.845**	0.057	0.007	**0.854**	0.052	−0.023	0.029	**0.812**	0.025	0.054
Talked to me often	**0.852**	−0.023	−0.043	**0.864**	−0.028	−0.077	0.038	**0.839**	−0.070	−0.018
Praised me	**0.809**	0.012	0.035	**0.814**	0.001	0.026	−0.061	**0.779**	−0.031	0.062
Let me do those things I liked doing	**0.498**	−0.063	**0.439**	**0.498**	−0.058	**0.429**	0.020	**0.311**	0.013	**0.629**
Tried to control everything I did	−0.049	**0.601**	−0.147	−0.049	**0.625**	−0.183	0.243	−0.012	**0.592**	−0.172
Invaded my privacy	−0.056	**0.686**	−0.045	−0.058	**0.710**	−0.079	0.247	−0.050	**0.688**	−0.049
Tended to baby me	**0.373**	**0.730**	0.001	**0.357**	**0.719**	0.034	−0.158	**0.371**	**0.700**	−0.008
Tried to make me dependent on him	0.093	**0.769**	0.035	0.080	**0.773**	0.040	0.029	0.063	**0.781**	0.066
Felt I could not look after myself unless he was around	0.014	**0.791**	0.047	−0.001	**0.794**	0.056	0.017	−0.009	**0.799**	0.062
Was overprotective of me	**0.321**	**0.696**	−0.118	0.309	**0.682**	−0.100	−0.111	**0.351**	**0.660**	−0.145
Liked me to make my own decisions	**0.349**	−0.242	**0.438**	**0.356**	−0.206	**0.382**	0.320	0.128	−0.111	**0.688**
Wanted me to grow up	0.056	−0.087	**0.244**	0.063	−0.035	0.184	**0.407**	−0.084	0.014	**0.415**
Let me decide things for myself	0.245	−0.288	**0.448**	0.246	−0.261	**0.411**	0.216	0.030	−0.171	**0.678**
Gave me as much freedom as I wanted^a^	−0.013	0.026	**0.926**	−0.024	0.033	**0.934**	−0.006	−0.084	−0.013	**0.734**
Let me go out as often as I wanted^a^	−0.047	0.029	**0.938**	−0.062	0.034	**0.955**	−0.041	−0.090	−0.032	**0.691**
Let me dress in any way I pleased^a^	0.086	0.007	**0.695**	0.076	0.011	**0.706**	−0.032	−0.010	0.008	**0.625**
	Factor correlation									
	Factor 1	Factor 2	Factor 3	Factor 1	Factor 2	Factor 3	Factor 4	Care	Overprotection	Autonomy
Factor 1 (care)	1.000			1.000				1.000		
Factor 2 (overprotection)	−0.245	1.000		−0.233	1.000			−0.187	1.000	
Factor 3 (autonomy)	0.367	−0.344	1.000	0.404	−0.360	1.000		0.522	−0.434	1.000
Factor 4				0.048	−0.172	0.107	1.000			

*Note*. Values in bold are to suggest the corresponding factors.

a Residual variances were specified for three items: “gave me as much freedom as I wanted,” “let me go out as often as I wanted,” and “let me dress in any way I pleased.”

#### Measurement Invariance Across Parents and Offspring Gender

Six multiple-group ESEMs were specified (Table 4, m1-m6). Offspring ratings of their mothers and fathers were both included in the same model, with sons and daughters forming two separate groups. Hence, the tests of measurement invariance conducted here are based on two types of ratings (mothers vs. fathers) provided by two (sons vs. daughters) groups of offspring. The baseline model (m1) tests whether the factorial structure is consistent across groups of offspring ratings of their mothers or fathers, allowing parameters to be freely estimated across respondents and parents. The baseline model provided an excellent model fit (RMSEA: 0.04, TLI: 0.971, CFI: 0.966), supporting the configural invariance of the model. In the weak invariance model (m2), factor loadings were constrained equal across groups of offspring and parental ratings. The model fitted the data well (RMSEA: 0.033, TLI: 0.979, CFI: 0.977), and in comparison with Model 1, there was improvement in goodness of fit in terms of RMSEA, CFI, and TLI, indicating equal factor loadings across groups and types of parental ratings. In the strong invariance model (m3), the focus is the invariance of the item thresholds. In addition to constraining factor loadings equal, thresholds were held equal across daughters and sons, as well as across ratings of mothers and fathers. Model 3 fitted the data well, and showed minimal change in model fit indices, including RMSEA, CFI, and TLI, supporting the strong measurement invariance of the model. In Model 4 (m4), strict invariance was imposed by additionally holding residual variances constant across all groups of offspring and parental ratings. Again, this model fitted the data well, and showed improved goodness of fit in comparison with the strong invariance model (m3), supporting the strict invariance of the model. In Model 5, the variances and covariances of all factors were constrained to be equal across groups and types of parental ratings. This model again resulted in improved goodness-of-fit results, thus supporting the invariance of the latent variance and covariance matrix across groups of offspring and parental ratings. Finally, tests of the invariance of the latent means across groups of offspring and parental ratings (m6) resulted in a slight decrease in goodness of fit in comparison with Model 5, where latent means were freely estimated. Even though the decrease in fit remained minimal (less than 0.01 in RMSEA, TLI, and CFI), we decided to explore latent means differences given their substantive interest. We thus retained Model m5 as the final model. The invariant latent correlations were estimated as part of this model, as well as latent means across all daughters’ and sons’ ratings of their mothers and fathers, and are reported in Table 4.

**Table 4.** Summary of Goodness-of-Fit Statistics for ESEM Measurement Invariance Analysis.

	Model	*n*	χ^2^	*df*	Free parameter	Model of comparison	χ^2^ Difference test	*p* (*df*)	RMSEA	CFI	TLI
Configural invariance	m1	3,208	6872.021	1,902	642		NA	NA	0.040	0.971	0.966
Weak invariance	m2	3,208	5777.900	2,091	453	m1	490.271	0.000 (189)	0.033	0.979	0.977
Strong invariance	m3	3,208	6993.026	2,226	318	m2	820.107	0.000 (135)	0.037	0.972	0.972
Strict invariance	m4	3,208	6912.728	2,298	246	m3	364.053	0.000 (72)	0.035	0.973	0.974
Var–cov invariance	m5	3,208	5726.835	2,325	219	m4	178.175	0.000 (27)	0.030	0.980	0.981
Latent mean invariance	m6	3,208	7207.481	2,334	210	m5	580.267	0.000 (9)	0.036	0.972	0.973
MIMIC saturated	m7	2,153	5200.654	1,107	357				0.041	0.966	0.959
MIMIC invariant	m8	2,153	5760.336	1,275	189	m7	754.710	0.000 (168)	0.040	0.962	0.961
MIMIC null model	m9	2,153	6116.799	1,299	165	m7	1132.176	0.000 (192)	0.042	0.96	0.959
						m8	308.955	0.000 (24)			
Path model	m10	3,422	7162.506	2,466	280				0.033	0.973	0.974
Path model father	m11	3,420	2910.727	624	178				0.046	0.98	0.98
Path model mother	m12	3,422	2552.116	624	178				0.042	0.978	0.977

*Note*. ESEM = exploratory structural equation modeling; *df* = degrees of freedom; RMSEA = root mean square error of approximation; CFI = comparative fit index; TLI = Tucker–Lewis index; MIMIC = multiple indicators multiple causes; var–cov = variance–covariance.

Factor correlations (Table 5) showed that, for both mothers and fathers, care was positively correlated with autonomy but negatively correlated with overprotection. There was a high level of agreement across parenting characteristics of mothers and fathers (correlation coefficients were 0.519 for care, 0.666 for overprotection, and 0.808 for autonomy). This is consistent with the observed invariance of the factor variances–covariances.

**Table 5.** Factor Correlations and Means Based on Variance–Covariance Invariant Model (m5).

	Latent means (*SE*)					
	Mother care	Mother overprotection	Mother autonomy	Father care	Father overprotection	Father autonomy
Sons	0	0	0	**−0.532** (**0.028**)	**−0.455** (**0.031**)	−0.029 (0.029)
Daughters	0.049 (0.037)	0.063 (0.039)	**−0.251** (**0.040**)	**−0.209** (**0.038**)	**−0.154** (**0.040**)	**−0.368** (**0.042**)
Sons	**0.523** (**0.028**)	**0.455** (**0.031**)	0.029 (0.029)	0	0	0
Daughters	**0.572** (**0.039**)	**0.518** (**0.043**)	**−0.222** (**0.043**)	**0.314** (**0.039)**	**0.301** (**0.042**)	**−0.339** (**0.043**)
Sons	−0.049 (0.037)	−0.063 (0.039)	**0.251** (0.040)	**−0.572** (**0.039**)	**−0.518** (**0.043**)	**0.222** (**0.043**)
Daughters	0	0	0	**−0.258** (**0.030**)	**−0.217** (**0.029**)	**−0.117** (**0.027**)
Sons	**0.209** (**0.038**)	**0.154** (**0.040**)	**0.368** (**0.042**)	**−0.314** (**0.039**)	**−0.301** (**0.042**)	**0.339** (**0.043**)
Daughters	**0.258** (**0.030**)	**0.217** (**0.029**)	**0.117** (**0.027**)	0	0	0
	Factor correlations					
	Mother care	Mother overprotection	Mother autonomy	Father care	Father overprotection	Father autonomy
Mother care	**1.000**					
Mother overprotection	**−0.263**	**1.000**				
Mother autonomy	**0.464**	**−0.376**	**1.000**			
Father care	**0.519**	**−0.127**	**0.261**	**1.000**		
Father overprotection	**−0.165**	**0.666**	**−0.248**	**−0.263**	**1.000**	
Father autonomy	**0.283**	**−0.190**	**0.808**	**0.464**	**−0.376**	**1.000**

*Note. SE* = standard error. All values in bold were statistically significant at *p* < .05.

Across the set of models considered here, the latent means are constrained at zero in one group of offspring rating of one parent (e.g., sons’ ratings of their mothers) for identification purposes, allowing for the free estimation of the latent means for all other ratings (e.g., sons’ ratings of their fathers, and daughters’ ratings of both parents). This way, all freely estimated latent means directly represent deviations, in standard deviation units, from the referent latent mean constrained at zero. In order to more specifically assess latent means differences, Model m5 was thus reestimated four times, each time with a different set of latent means set to zero (the reference point). Examination of these results (see Table 5) suggests differences in parenting of mothers and fathers according both to sons and daughters. Compared with maternal measures, both daughters and sons rated fathers to be less caring (−0.532 for sons, −0.258 for daughters) and less overprotective (−0.455 for sons, −0.217 for daughters). Daughters also rated fathers as granting less autonomy (−0.117), although there was no difference for sons (−0.029, *ns*). There were also differences in how sons and daughters viewed the parenting of their mothers and fathers. There was no difference in how sons and daughters rated their mothers’ parenting styles on care and overprotection. However, in comparison with the sons’ perception of their mothers, daughters regarded their mothers as giving less autonomy (−0.251). Daughters also regarded fathers as more caring (0.314), more protective (0.301), and as granting less autonomy (−0.339) compared with sons.

#### DIF Analysis

In order to assess whether PBI ratings were subject to DIF in relation to covariates including personality and mental health measures, MIMIC ESEMs were estimated, starting from the model of strict measurement invariance (Model m4). These results are reported in Table 4. In comparison with Models m7 (MIMIC Saturated) and m8 (MIMIC Invariant),Model m9 (MIMIC null) resulted in almost identical goodness-of-fit indices, suggesting that these covariates had no effects on sons and daughters ratings of their parents, thus also evidencing a lack of DIF and measurement biases relate to these covariates.

### Predictive Validity of PBI Factors

Starting again from a model of strict measurement invariance (Model m4), we first estimated a multiple-group model with both maternal and paternal factors included as predictors of mental health outcomes at age 43 and 53 (Model m10, see Table 6). For daughters, none of the parenting style factors predicted mental health outcomes. However, for sons, paternal care predicted fewer mental health symptoms at age 43 (beta = −0.139). It is noteworthy that, although none of the predictions came out as significant in the daughters group, this group evidenced some inflated standardized regression coefficients and standard errors, suggesting the presence of multicollinearity among parenting style measures related to mothers and fathers. This implies that for parenting related to overprotection and autonomy, there was limited unique contribution of maternal or paternal parenting effects on later life mental health

**Table 6.** Mental Health Outcomes at Ages 43 and 53 Predicted by PBI Factors (Model m10).

	Mental health outcomes	
	Age 43	Age 53
*Male offspring*		
Maternal care up to age 16	0.080	0.008
Maternal overprotection up to age 16	0.085	0.018
Maternal autonomy up to age 16	−0.130	−0.042
Paternal care up to age 16	**−0.139**	−0.029
Paternal overprotection up to age 16	0.005	0.013
Paternal autonomy up to age 16	0.025	−0.029
Mental health age 43		**0.354**
*Female offspring*		
Maternal care up to age 16	−0.140	−0.094
Maternal overprotection up to age 16	0.063	0.169
Maternal autonomy up to age 16	0.146	0.280
Paternal care up to age 16	0.000	0.055
Paternal overprotection up to age 16	0.080	−0.038
Paternal autonomy up to age 16	−0.100	−0.267
Mental health age 43		**0.326**

*Note*. PBI = Parental Bonding Instrument. Regression paths are presented in standardized metric. Values in bold are statistically significant at *p* < .05. The measurement specification is based on multiple-group analysis for sons and daughters with strict invariance specification. Mental health outcome at age 53 was adjusted for mental health outcome at age 43. The results are adjusted for occupational social class at ages 11 and 43.

## Discussion

The present study is the first psychometric investigation of PBI based on a large representative population-based sample from the United Kingdom. Analyses supported a three-factor structure in the study population, and multiple-group ESEM and MIMIC models demonstrated the robustness of the psychometric properties of the PBI instrument as a function of the respondents’ and parents’ genders, as well as respondents’ personality characteristics and mental health. The PBI was found to predictive of mental health in midlife in a gender-specific manner.

### Psychometric Properties of the PBI

ESEM analyses of the NSHD sample led to a three-factor structure (Tables 1, 2, and 3). The care factor corresponded to the original factor of this name (Parker, 1979), but the control factor split into two further factors, overprotection and autonomy, in line with some psychometric studies of Western samples (Cox et al., 2000; Heider et al., 2005; E. Murphy et al., 1997). Although previous studies in non-Western cultures have found that a four-factor structure explained the data better (Behzadi & Parker, 2015; Liu et al., 2011; Suzuki & Kitamura, 2011; Uji et al., 2006), the three-factor solution found in the present investigation is consistent with several previous studies of English-speaking populations or other Western cultures (Cubis et al., 1989; Gómez-Beneyto et al., 1993; Kendler, 1996; Mohr, Preisig, Fenton, & Ferrero, 1999; E. Murphy et al., 1997). Countries in which the our factor solution was supported, such as Iran or Asian countries, are often characterized by a male-dominated culture in familial and societal environments, which may lead to differences in parenting styles when assessed using an instrument initially developed for Western cultures (Behzadi & Parker, 2015). Another possibility is the effect of cultural differences on the individual’s response to the wordings of questionnaire items. The items that form further additional factors of the PBI are often items that score in the same direction toward either positive parenting or negative parenting. It has been suggested that responses to questionnaire items with mixed wordings (positive and negative) can be subject to cultural influences. In particular, negatively worded items are often interpreted differently across cultures (Schmitt & Allik, 2005). It has been suggested that additional factors due to positive/negative wording of items are interpreted as artefactual (Greenberger, Chen, Dmitrieva, & Farruggia, 2003).

Our study is the first to demonstrate the measurement invariance of the PBI (m1-m6, Table 4) in relation to parental and offspring gender, utilizing sophisticated psychometric methods to verify the factor structure and measurement invariance across distinct gender groups. Under multiple-group ESEM analysis, the three-factor structure in the current sample was found to be fully invariant in these respects, which enables valid comparison of analyses involving maternal and paternal parenting practices, and in male and female offspring.

Both male and female participants rated their fathers to be less caring and less overprotective than their mothers, which is in line with previous findings that mothers tend to adopt warmer parenting styles compared with fathers (E. Murphy et al., 1997; Russell et al., 1998). This is in line with the gender role theory that women are socialized to be warmer and more caregiving compared with their male counterparts, whereas men are perceived as more authoritarian (Hosley & Montemayor, 1997). However, this result may potentially also reflect cohort effects, as participants from the present study were born in the 1940s.

The psychometric properties of the PBI ratings were also shown to be robust in relation to external covariates (Table 4, m7-m8). This finding lends stronger support to the validity of associations reported in previous studies investigating the relations between parental styles and personality/mental health outcomes. Although previous studies also looked at the potential bias of PBI ratings due to factors such as concurrent depressive mood (Duggan, Sham, Minne, Lee, & Murray, 1998; Gotlib, Mount, Cordy, & Whiffen, 1988; Rodgers, 1996a also based on the NSHD sample), the present study is the first to rely on a psychometric approach that allows for the assessment of DIF in relation to individual items. This makes possible a more comprehensive examination of the measurement properties in relation to key covariates that are often studied as outcomes of parenting styles.

### Predicative Validity of PBI Factors

In the present investigation, we did not find a unique effect of maternal or paternal parenting style factors for mental health outcomes in daughters. However, fathers’ care predicted fewer mental health symptoms in sons. Although we are not able to find other studies with follow-up going well into adulthood, Lansford et al. (2014) reported the unique effect of father’s autonomy on sons’ externalizing behaviors. An earlier review (Pleck & Masciadrelli, 2004) also showed evidence of the father’s involvement in longer term child outcomes. This might be explained from the perspective of role theory (Hosley & Montemayor, 1997) that sons are traditionally encouraged to be more independent and take more risks. Indeed, children tend to copy behavior from parents of the same sex (Laible & Carlo, 2004). Here, the results suggest that caring parenting from fathers predicts positive mental health in sons.

The lack of a unique parental style association for daughters is likely due to substantial confounding of maternal and paternal measurements, given the high correlation among maternal and paternal measures observed in the present study (*r* = 0.519-0.808). The separate results of maternal and paternal predictions (Table S5) confirmed that the gender-specific effect of the PBI was highly comparable for both sons and daughters. Studies with adolescents have also reported a moderate to high level of concordance between perceptions of mothers and fathers (Hoeve et al., 2011; Lansford et al., 2014). This indicates that it might be sufficient to measure PBI from one rather than from both parents.

### Limitations and Future Directions

Sample attrition was found to relate to gender and occupational social class, indicating the missing at random mechanism. To account for this limitation, the analyses included occupational social class as a covariate, and the analyses were also stratified by gender. The WLSMV estimator used in the current analysis employs a pairwise-present strategy for dealing with missing data, which has been shown to produce unbiased estimates under missing at random assumptions in relation to observed covariates (Asparouhov & Muthén, 2010).

A feature of the current study design is that the parenting styles for prior to the age of 16 years were assessed while the participants were 43 years old. Although our study is unable to assess test-retest stability, as it is limited to only one wave of measurement, previous recollections of parenting styles have been shown to remain stable even when reassessed after a 20-year period (Eleanor Murphy et al., 2010; Wilhelm, Niven, Parker, & Hadzi-Pavlovic, 2005). Nevertheless, it would be interesting to study how parenting styles measured at different stages predict future outcomes.

The current study is based on a British sample for which English is the native language. Since the PBI has also been studied across a diverse range of countries (Spain: Gómez-Beneyto et al., 1993; China: Liu et al., 2011; Australia: Mackinnon et al., 1989; France: Mohr et al., 1999; E. Murphy et al., 1997; Pakistan: Qadir, Stewart, Khan, & Prince, 2005; Brazil: Terra et al., 2009; Japan: Uji et al., 2006), it would be interesting to assess whether this property studied in the current study holds true for other cultures.

Another limitation of the current study is that all participants were White, therefore future cross-cultural studies are needed to address the lack of cultural diversity in the current sample.
